# Sudden sensorineural hearing loss as the initial symptom in patients with acoustic neuroma

**DOI:** 10.3389/fneur.2022.953265

**Published:** 2022-08-17

**Authors:** Mengtao Song, Dayong Wang, Jin Li, Guohui Chen, Xiaolong Zhang, Hongyang Wang, Qiuju Wang

**Affiliations:** ^1^College of Otolaryngology, Head and Neck Surgery, Institute of Otolaryngology, Chinese People's Liberation Army General Hospital, Beijing, China; ^2^National Clinical Research Center for Otolaryngologic Diseases, Beijing, China

**Keywords:** acoustic neuroma, sudden sensorineural hearing loss, pure-tone audiometry, auditory brainstem response, magnetic resonace imaging

## Abstract

**Background:**

Previous studies have shown that patients with acoustic neuroma (AN) sometimes present with sudden sensorineural hearing loss (SSNHL) as an initial symptom. The purpose of this research was to investigate the clinical characteristics, diagnosis, and treatment of AN in patients initially diagnosed with SSNHL.

**Materials and methods:**

We reviewed retrospectively the medical records of all patients who were treated as SSNHL initially and were later diagnosed with AN after undergoing magnetic resonance imaging (MRI) at our hospital between 2008 and 2021. Patient demographics, associated complaints (mostly tinnitus and vertigo), the severity of hearing loss, audiogram configurations, auditory brainstem response (ABR), and MRI examination were reviewed and analyzed. In addition, treatment outcomes and management protocols were also included in this study.

**Results:**

A total of 10 (0.7%, 10/1,383) patients presented with SSNHL as the initial symptom and were diagnosed as AN by MRI finally. Of the 10 patients enrolled in this study, four were men and six were women. The average age at the time of diagnosis of SSNHL was 46.2 ± 13.16 years. These patients exhibited varying severity of hearing loss and a variety of audiogram configurations. All patients showed an abnormal ABR. According to the Koos grading standard, there were 5 grade I (intracanalicular [IAC]) tumors, 3 grade II tumors, and 2 grade III tumors. The treatment outcome revealed that 2 patients exhibited recovery of the average hearing of impaired frequency by more than 15 dB, and 6 patients showed no recovery. Furthermore, four patients were referred to undergo surgical treatment after being diagnosed with AN, 1 patient accepted stereotactic radiation therapy, and the remaining 5 patients were on a “wait and scan” strategy.

**Conclusion:**

The hearing loss of patients with AN presented with SSNHL may improve with drug treatment. Hearing recovery for SSNHL does not exclude the presence of AN, and all patients initially diagnosed with SSNHL should undergo MRI and ABR to prevent misdiagnosis and delays in potential treatment.

## Introduction

Sudden sensorineural hearing loss (SSNHL) is defined as a rapid-onset sensorineural hearing loss of ≥20 decibels (dB), affecting at least 2 consecutive frequencies that occurred within 72 h with no identifiable cause (1). The incidence of SSNHL is reported to be 5–20 per 100,000 individuals, with about 66,000 new cases per year in the United States (2). An epidemiological survey in Japan revealed that the incidence of SSNHL was 60.9 per 100,000 population (3). In China, the prevalence of SSNHL has been on the rise in recent years, but large samples of epidemiological data are lacking. For patients suffering from SSNHL, more than 90% of cases are idiopathic and the remainder is due to causes such as acoustic neuroma, stroke, malignancy, Meniere's disease, trauma, autoimmune disease, syphilis, Lyme disease, and perilymphatic fistula (4, 5).

Acoustic neuroma (AN) is known as a benign tumor that originates from the superior or inferior vestibular branch of the cochleovestibular nerve within the internal auditory canal (IAC) and grows into the cerebellopontine angle (CPA) (6). Sensorineural hearing loss is the major presentation of patients with AN, and patients with AN can present with SSNHL as an initial symptom occasionally (7–9). According to previous studies, the reported prevalence of AN in patients presenting with SSNHL ranges from 1.8 to 5.2% (10, 11). As a result of the increasingly widespread use of magnetic resonance imaging (MRI), more patients with AN than expected have been detected among those with SSNHL. MRI is regarded as the gold standard for imaging diagnosis of AN. In the meantime, the auditory brainstem response (ABR) test is recommended for the initial assessment of patients with SSNHL when appropriate, and it is highly sensitive to AN larger than 10 mm in size (10). AN is the most frequently observed MRI abnormality in patients with SSNHL (9). According to the American Academy of Otolaryngology-Head and Neck Surgery (AAO-HNS) guidelines, MRI or ABR should be used for the retrocochlear pathology evaluation of patients with SSNHL (2).

Previous studies have found that hearing can improve with corticosteroid treatment in patients with AN initially presenting with SSNHL (12–14) and that drug therapy is usually administered before MRI is performed. As a result, some physicians may assume that patients with SSNHL who respond to steroid therapy could effectively exclude the presence of AN, which leads to delays in the diagnosis. Since a small number of patients with SSNHL whose hearing loss is caused by AN, which is also prone to clinical misdiagnosis and missed diagnosis.

In the present study, we conducted a retrospective study of 10 patients with AN presenting with SSNHL as an initial symptom, with the aim of clarifying the detailed clinical features of the disease and the efficacy of treatment, and guiding clinicians to prevent misdiagnosis.

## Methods

### Study design and participants

We reviewed retrospectively the medical records of all patients admitted to our hospital between 2008 and 2021 who were initially treated as SSNHL and were later diagnosed as AN after undergoing MRI. All patients enrolled in the study underwent a targeted MRI scan of the brain and were treated for at least 7 days with drug therapy. These patients met the clinical diagnostic criteria for SSNHL, which is defined as a sensorineural hearing loss of 20 dB or more over at least 2 contiguous frequencies that develops within 3 days (1). Data collection included patient demographics, associated complaints (mostly tinnitus and vertigo), results of pure-tone audiometry (PTA), ABR, and MRI examination. Exclusion criteria included known previous or progressive hearing loss, AN that had been diagnosed before SSNHL occurred, and another inner-ear disease. Patients who did not undergo MRI examination were also excluded.

### Audiological assessment

All patients received the essential audiological examinations, such as pure-tone audiometry (GSI-61 dual channel diagnostic audiometer), speech recognition score (detected *via* GSI-61 clinical audiometer, with the acoustic stimulus of speech signal), and ABR. The severity of hearing loss was classified based on the criteria of the World Report on Hearing published by the World Health Organization (WHO) in 2021 (15). According to this criterion, the severity of the hearing loss is categorized into 7 grades based on hearing thresholds measured with PTA at 0.5, 1, 2, and 4 kHz:

1. Normal Hearing, the Mean Hearing Threshold 20 dB;
2. Mild Hearing Loss, the Mean Hearing Threshold Is 20 to < 35 dB;
3. Moderate Hearing Loss, the Mean Hearing Threshold Is 35 to < 50 dB;
4. Moderately Severe Hearing Loss, the Mean Hearing Threshold Is 50 to < 65 dB;
5. Severe Hearing Loss, the Mean Hearing Threshold Is 65 to < 80 dB;
6. Profound Hearing Loss, the Mean Hearing Threshold Is 80 to < 95 dB;
7. Complete or Total Hearing Loss/Deafness, the Mean Hearing Threshold Is 95 dB or Greater.

We also analyzed the patterns of hearing loss and the configuration of audiogram was categorized into 7 forms: low-frequency ascending form, U-shaped form, high-frequency descending form, flat form, profound form, dip form, and other forms (16) (as shown in Supplementary Table 1).

Results of the ABR were considered abnormal when they met at least one of the following criteria: (1) absent evoked response upon the compatible auditory threshold; (2) desynchronization of waves other than wave I; (3) interpeak latency (IPL) between waves I and III > 2.5 ms; (4) IPL between waves I and V > 4.4 ms; (5) Wave V interaural latency difference (ILD) > 0.2 ms; and (6) interaural difference of IPL between waves I and V > 0.2 ms (17).

### Imaging examination

MRI was performed to make a definite diagnosis of AN and to rule out other explanations for hearing loss. We used either a 1.5-T or a 3.0-T magnet for MRI examination. The scans included high-resolution T2 sequences and contrast-enhanced T1-weighted MRI directed to the IAC and cerebellopontine angle (CPA). The images were reviewed by experienced radiologists and otologists. The tumor size was measured on MRI images, and the type of tumor within the intracanalicular (IAC) and the maximum diameter of the tumor in CPA were used according to the recommendation of the Summary and consensus in the 7th International Conference on Acoustic Neuroma (6). According to the Koos grading standard, the tumor size is classified as follows: grade I, tumor is confined to the IAC and the maximum diameter is ≤ 1 cm; grade II, small tumor protrusion into CPA without contact with the brain stem, diameter ranges from 1.1 to 2 cm; grade III, tumor occupying the CPA with no brainstem displacement, diameter ranges from 2.1 to 3 cm; and grade IV, large tumor with brainstem and cranial nerve displacement, diameter is more than 3.0 cm (18).

### Treatment evaluation

During the hospitalization, all patients enrolled in the study were treated for 1 or 2 weeks with the same corticosteroid treatment protocol (first, dexamethasone is administered intravenously for the first 3 days with 10 mg/day and for the next 3 days with 5 mg/day, and then it is changed to methylprednisolone, which is administered as a postaural injection, 40 mg, one time for every 3 days). The initial hearing level was determined by the first audiometric evaluation before treatment and the final hearing level was tested 2–4 weeks after treatment. The evaluation of the treatment outcome was based on the hearing recovery criteria described in the Guidelines for the diagnosis and treatment of SSNHL (published in China in 2015) (1):

1. Complete recovery: the hearing thresholds of impaired frequency returned to normal, or reached the level of contralateral ears or the original level.
2. Significant recovery: the hearing thresholds of impaired frequency increased ≥30 dB on average.
3. Slight recovery: the hearing thresholds of impaired frequency increased ≥15 dB and < 30 dB on average.
4. No recovery: the average hearing threshold of impaired frequency increased < 15 dB.

We evaluated the rates of hearing recovery in the study groups and a summed rate of complete recovery, significant recovery, and slight recovery was defined as the “effective” rate.

### Statistical analysis

Data analysis was performed with GraphPad Prism 8.3.0 (2019, GraphPad Software, LLC., USA) and Microsoft Office Excel 2019 (Office, Microsoft, USA). Continuous variables were summarized using means, standard deviations (SDs), and range values when normally distributed; categorical data were summarized as numbers (percentage) and analyzed by the χ^2^ or Fisher's tests when normally distributed. Spearman's rank correlation analysis was used to determine the relationship among the investigated factors, and the results were presented with a confidence interval (*CI*) of 95%. A *p-*value of < 0.05 was considered statistically significant.

## Results

### Patients

In total, 1,383 patients with SSNHL who underwent MRI were identified, and 10 (0.7%) patients were finally diagnosed as AN. The demographic characteristics of the patients are summarized in Table 1. There were 4 men and 6 women, ranging in age from 26 to 70 years during the diagnosis of SSNHL with a mean age of 46.2 ± 13.16 years. Of the 10 patients enrolled in this study, 9 showed a tumor on the same side of SSNHL, with 4 on the left side and 5 on the right. There was one patient with SSNHL in the bilateral ear who had an incidental finding of AN in the right ear (case 8). Among the 10 patients, 9 (90%) patients complained of tinnitus and 2 patients (20%) complained of dizziness as an accompanying symptom. Three patients underwent vestibular function examination with 1 patient showing a reduced function of the left horizontal semicircular canal, 1 patient showing a reduced function of the right horizontal semicircular canal, and 1 patient with a positive positional test.

**Table 1:** The clinical characteristics of patients with sudden sensorineural hearing loss (SSNHL) and acoustic neuroma (AN).

**Case no**.	**Gender**	**Age**	**SNHL side**	**Tumor side**	**Tinnitus**	**Dizziness**	**Configuration of audiograms**	**PTA (dB)**	**Severity of hearing loss**	**Recovery of hearing**	**Tumor size (cm)**	**Koos Grade**	**Treatment**
1	F	38	L	L	+	—	U-shape	67.5	Severe	Significant	1.4	II	Wait and scan
2	M	38	L	L	—	—	High-frequency descending	21.25	Mild	/	1.0	I	Wait and scan
3	F	49	R	R	+	—	U-shape	72.5	Severe	No recovery	2.7	III	Surgery
4	F	59	R	R	+	+	Profound	120	Deafness	/	0.7	I	SRT
5	F	45	R	R	+	—	Other	68.75	Severe	No recovery	2.5	III	Surgery
6	M	31	R	R	+	—	Dip	33.75	Mild	Slight	1.5	II	Surgery
7	F	70	L	L	+	+	Flat	67.5	Severe	No recovery	1.2	I	Surgery
8	M	45	Bilateral	R	+	—	High-frequency descending	43.75	Moderate	No recovery	0.9	I	Wait and scan
9	F	61	R	R	+	—	Flat	93.75	Profound	No recovery	1.5	II	Wait and scan
10	M	26	L	L	+	—	High-frequency descending	18.75	Normal	No recovery	1.0	I	Wait and scan

SNHL, Sudden sensorineural hearing loss; AN, Acoustic neuroma; F, Female; M, Man; L, Left; R, Right; PTA, Pure tone threshold; SRT, Stereotactic radiation therapy.

### Audiology

#### The severity of hearing loss

Among the 10 patients, the severity of hearing loss of the affected ear was characterized as mild in 2 ears (20%), moderate in 1 ear (10%), severe in 4 ears (40%), profound in 1 ear (10%), and deafness in 1 ear (10%). In addition, one patient presented a normal hearing threshold at frequencies of 0.25, 0.5, 1, and 2 kHz, with 45 dB hearing loss at 4 kHz. The PTA ranged from 18.75 to 120 dB, with a mean range of 60.75 ± 30.39 dB.

#### Audiogram configuration

**Figure 1:**
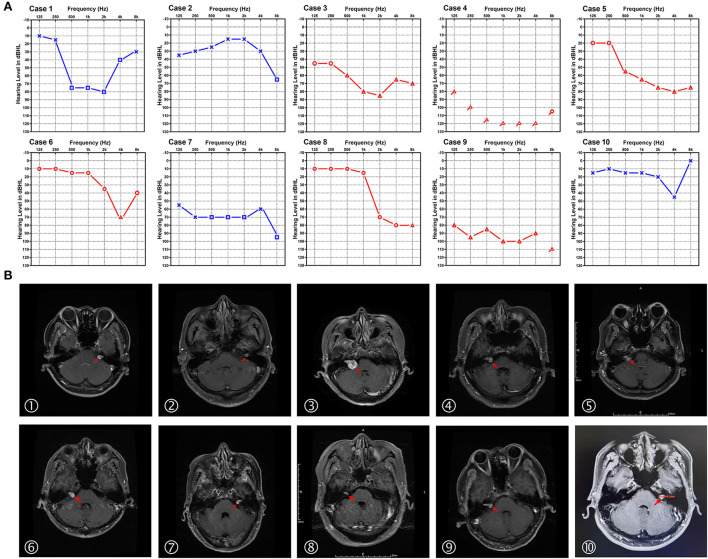
Audiograms and MRI image of the 10 patients with sudden sensorineural hearing loss (SSNHL) and acoustic neuroma (AN). **(A)** Pure-tone audiograms of the affected ear of the 10 patients with acoustic neuroma at the diagnosis of sudden sensorineural hearing loss (air conduction). The red line represents the right ear and the blue line represents the left ear. **(B)** The axial cranial MRI of the 10 patients diagnosed with acoustic neuroma in our study. Red arrows indicate the tumor location.

The audiograms of the affected ears of all 10 patients when diagnosed with SSNHL are depicted in Figure 1A. Audiogram configurations were analyzed and for the 7 forms of audiogram configurations, 2 ears (20%) were classified as U-shape, 3 (30%) as high-frequency descending, 2 (20%) as flat, 1 (10%) as dip, 1 (10%) as profound, and 1 (10%) as other.

#### Auditory brainstem response

As for ABR examinations, all 10 patients were evaluated as having an abnormal ABR according to the diagnostic criteria mentioned above. The sensitivity of ABR for the detection of AN was 100% in our study. Table 2 demonstrated the latency of ABR waves of the 10 enrolled patients. In these patients who showed an abnormal ABR waveform pattern, no response was detected in 2 patients (20%) at 100 dB SPL, 2 patients (20%) had only wave V present, 3 patients (30%) showed the absence of wave I, 2 patients (20%) with IPL between waves I and III > 2.5 ms, 4 patients (40%) with IPL between waves I and V > 4.4 ms. Significantly, except for 2 patients with no response, the ILD of wave V were all > 0.2 ms in the remaining 8 patients, with a mean value of 1.01 ± 0.43 ms.

**Table 2:** The latency of ABR waves of the 10 patients enrolled in our study.

**Case no**.	**Tumor side**	**Side**	**wave I** **(ms)**	**wave III** **(ms)**	**wave V** **(ms)**	**wave I-III** **(ms)**	**wave III-V** **(ms)**	**wave I-V (ms)**	**ILD (ms)**
1	L	L	1.92	4.48	6.33	2.56	1.84	4.40	1.23
		R	1.48	3.56	5.10	2.08	1.54	3.62	
2	L	L	1.25	/	6.38	/	/	5.03	1.06
		R	1.11	/	5.32	/	/	4.21	
3	R	L	1.50	3.92	5.50	2.42	1.58	4.00	1.65
		R	NR	5.33	7.15	NR	1.82	NR	
4	R	L	1.65	3.95	5.68	2.30	1.73	4.03	/
		R	NR	NR	NR	NR	NR	NR	
5	R	L	1.33	3.40	5.43	2.07	2.03	4.10	1.55
		R	NR	NR	6.98	NR	NR	NR	
6	R	L	1.55	3.65	5.55	2.10	1.90	4.00	0.55
		R	2.08	4.20	6.10	2.12	1.90	4.02	
7	L	L	1.59	3.94	6.48	2.35	2.54	4.89	0.87
		R	1.14	3.62	5.61	2.48	1.99	4.47	
8	R	L	1.50	3.68	5.58	2.18	1.90	4.08	0.80
		R	NR	NR	6.38	NR	NR	NR	
9	R	L	1.68	3.98	5.85	2.30	1.87	4.17	/
		R	NR	NR	NR	NR	NR	NR	
10	L	L	1.63	4.05	6.05	2.42	2.00	4.42	0.35
		R	1.55	3.85	5.70	2.30	1.85	4.15	

ABR, Auditory brainstem response; L, Left; R, Right; ILD, Interaural latency difference; NR, No response.

### Imaging findings and correlation

**Figure 2:**
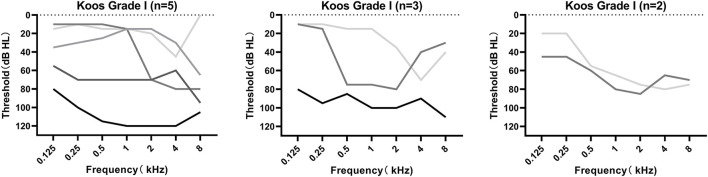
Audiograms of the 10 patients with acoustic neuroma diagnosed as a sudden sensorineural hearing loss initially classified according to the Koos grading system. Audiometry found no consistent trend with regard to the Koos grading system.

MRI was performed in all 10 patients, revealing tumors ranging from 0.7 to 2.7 cm, with a mean size of 1.44 ± 0.63 cm. Figure 1B demonstrates the axial cranial MRI of these patients. According to the Koos grading standard, there were 5 grade I (intracanalicular) tumors, 3 grade II tumors, and 2 grade III tumors. The audiograms of patients with grades I–III tumors are shown in Figure 2. There was no obvious relation between tumor size and hearing loss with regard to the audiometric pattern. In addition, we conducted correlation analyses between tumor size and grade of hearing loss and the configuration of audiograms. There was no significant correlation between tumor size and grade of hearing loss (*r* = 0.1136, *p* = 0.7533; Spearman's rank correlation test). Tumor size and configuration of audiograms were also unrelated (*r* = 0.0528, *p* = 0.8831; Spearman's rank correlation test).

### Management results

**Figure 3:**
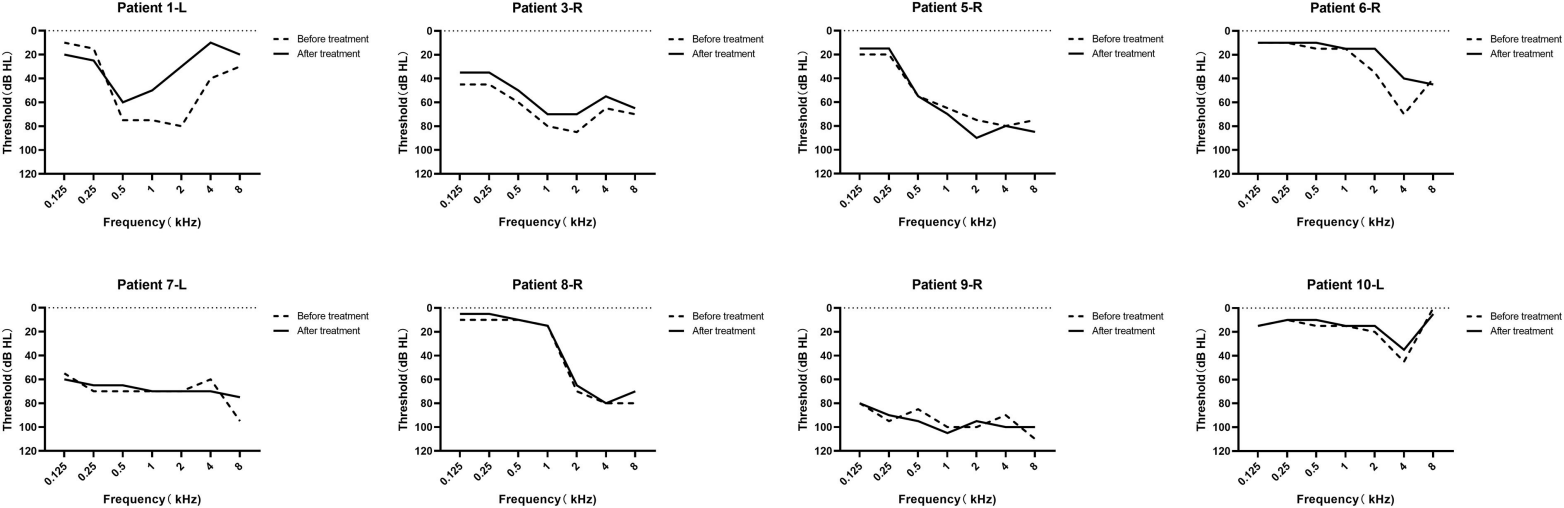
Audiograms of the eight patients who recorded completed pure tone audiometry (PTA) results before and after drug treatment. The “L” and “R” means the tumor side. The dashed line represents the hearing threshold before treatment, and the solid line represents the hearing threshold after treatment.

**Figure 4:**
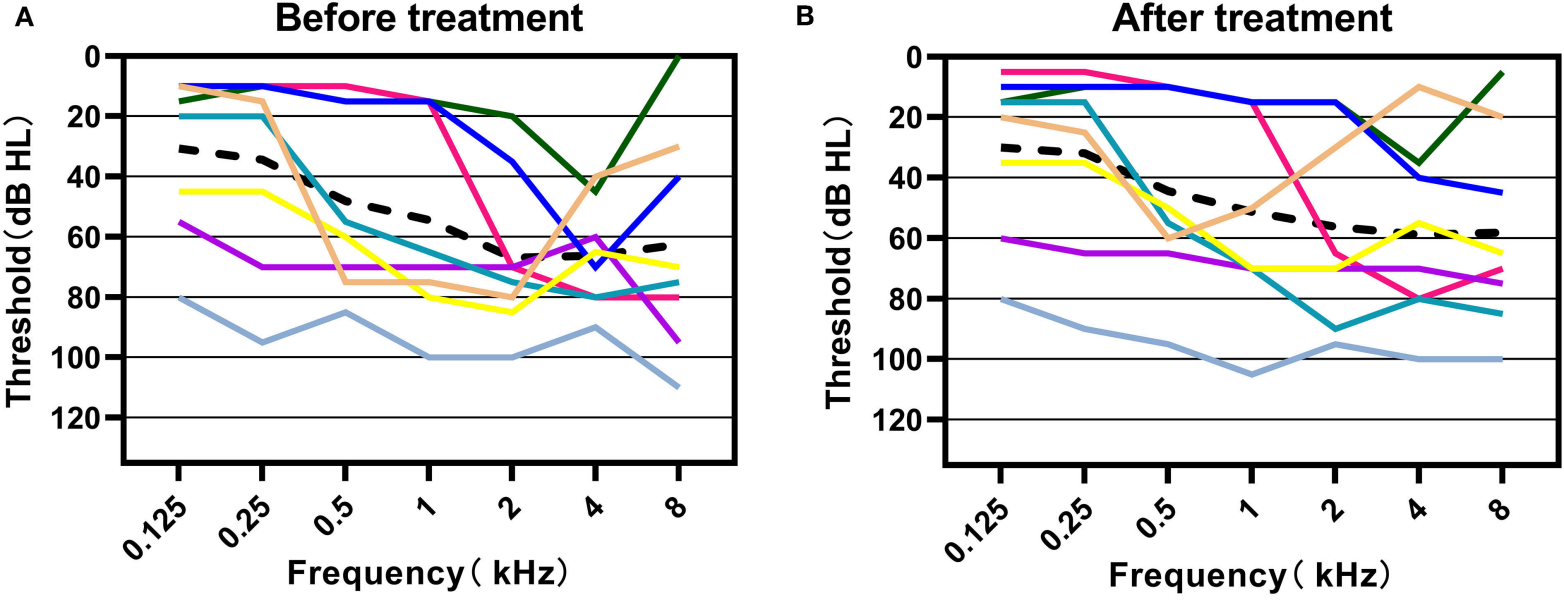
Hearing thresholds before and after the treatment of these patients in our study. **(A)** Pure-tone thresholds at the time of diagnosis of sudden sensorineural hearing loss in all 10 patients. **(B)** Pure-tone thresholds after drug treatment of sudden sensorineural hearing loss in 8 patients who recorded completed pure tone audiometric results. The black dashed line represents the average hearing thresholds at each frequency.

Among the 10 patients in our study, 8 recorded detailed PTA results before and after drug treatment (Figure 3), while the other 2 patients had either subjective report only or incomplete audiometric data available. The treatment outcome revealed that 2 (25%) of the 8 patients exhibited recovery of the average hearing of impaired frequency by more than 15 dB, and 6 (75%) patients showed no recovery. Figure 4 illustrates pure tone thresholds of the 8 patients who recorded detailed PTA results at the diagnosis of SSNHL and after drug treatment. Overall, there was no obvious recovery before and after drug treatment. In addition, we followed up on the speech recognition score (SRS) of 4 patients after drug treatment, and 2 of them showed significant improvement in the SRS compared with that before treatment: case 6 showed an increase in SRS from 68 to 100%, and case 7 showed a significant increase from 8 to 96%. For the final management of the 10 patients, 4 patients were referred to undergo surgical treatment after being diagnosed with AN, 1 patient accepted stereotactic radiation therapy, and the remaining 5 patients were given a “wait and scan” strategy (observation and follow-up of MRI). Among the 4 patients with surgical treatment, 3 patients were performed with a translabyrinthine approach and 1 with a retrosigmoid approach, and the postoperative hearing of the affected ear of the 4 patients was all totally deafness (as shown in Supplementary Figure 1).

## Discussion

Acoustic neuroma is the most common posterior cranial fossa tumor in adults, accounting for 6–8% of all intracranial tumors and more than 80% of tumors in the CPA (19). The early symptoms in patients with AN are mainly unilateral sensorineural hearing loss, tinnitus, and vertigo (20). SSNHL occurs in 10–20% of patients with AN at some point in their medical history, but the incidence of AN is somewhat lower in patients with SSNHL (4). We summarized previous studies where AN was detected in patients with SSNHL in Table 3 and the prevalence ranges from 1.8 to 5.2%. In our study, the incidence of AN in patients with SSNHL was 0.7% (10/1383). Sensorineural hearing loss is the major presentation of patients with AN and is often accompanied by tinnitus. In the present study, 90% (9/10) of patients with AN who presented SSNHL as an initial symptom and complained of tinnitus, which is consistent with the incidence of tinnitus in patients with AN (51–92%) reported in previous studies (32–34). Even though tinnitus is a nonspecific symptom, clinicians should still be alert to patients with unilateral SSNHL and tinnitus to avoid missing the diagnosis. Meanwhile, the absence of accompanying symptoms does not eliminate the possibility of a tumor.

**Table 3:** Summary of studies where acoustic neuroma (AN) was detected in a sudden sensorineural hearing loss (SSNHL).

**Studies**	**Incidence of AN in SSNHL**
Our study	0.7% (10/1,383)
Saunders et al. (20)	1.5% (13/836)
Aslan et al. (21)	4.2% (8/192)
Daniels et al. (22)	3.7% (2/54)
Fitzgerald and Mark. (23)	3.8% (3/78)
Aarnisalo et al. (24)	4.9% (4/82)
Cadoni et al. (25)	1.9% (1/54)
Suzuki et al. (26)	2.6% (13/500)
Lee et al. (13)	4.1% (12/295)
Lin et al. (27)	1.8% (10/542)
Jeong et al. (9)	3.1% (9/291)
Cho et al. (28)	5% (10/200)
Califano et al. (11)	5.2% (5/96)
Hosokawa et al. (29)	2.4% (20/848)
Fujita et al. (30)	3% (15/499)
Ungar et al. (31)	4.8% (20/420)
Yang et al. (10)	1.1% (14/1,249)

The audiogram configuration of SSNHL can affect high, low, or all frequencies, and audiograms are generally classified into low-frequency ascending, high-frequency descending, flat-type, and profound hearing loss (1). Previous studies have shown that the audiogram configuration may be trough-shaped or U-shape in patients with AN who are presenting with SSNHL as a primary symptom (7, 32). Furthermore, a recent study has found that the trough-shaped or U-shape audiogram was significantly more prevalent in patients with AN than in patients with ordinary idiopathic SSNHL, and the incidence of AN in SSNHL patients with trough-shaped or U-shape audiogram was significantly higher in SSNHL patients with other audiogram configurations (26, 29). This study suggests that a trough-shaped or U-shape audiogram in patients with SSNHL may indicate the presence of AN. In our study, the incidence of U-shape audiograms (20%, 2/10) in SSNHL patients with AN was not significantly specific compared with other configurations, which may be due to the small sample size.

As the gold standard for AN diagnosis, MRI is the preferred examination and can provide exquisite tumor characterization, surgical planning, and post-therapeutic assessment (19). High-resolution MRI can detect tumors smaller than 1 cm located in the IAC and differentiate AN from other masses, such as facial nerve schwannoma, meningioma, epidermoid cyst, arachnoid cyst, aneurysm, and metastasis (28, 35). According to previous studies, AN can be successfully diagnosed and largely differentiated from other lesions with 96–100% sensitivity and 88–93% specificity with the combination of T1- and T2-weighted MRI (36). In the present study, all the enrolled patients with SSNHL underwent contrast-enhanced MRI and 10 patients were diagnosed as AN. MRI is considered an excellent noninvasive evaluation for CPA lesions (acoustic neuroma, meningioma, trigeminal schwannoma, epidermoid cysts, etc.). The high contrast resolution and multiplanar capabilities of MR help to identify the site and extension of the lesions as well as the characteristic signal. A recent study found that CPA lesions were detected with 90% sensitivity and 99.5% specificity on high-resolution T2-weighted MRI compared with T1-weighted MRI with contrast (37).

In addition to MRI, ABRs have been used widely as a screening procedure for the diagnosis of AN, particularly when MRI is not available. In our study, abnormal ABR results were obtained in all patients, and the overall ABR sensitivity in diagnosing AN in SSNHL was 100%. Due to the small sample size, our results do not indicate the sensitivity of the ABR. We have known that ABR testing has limits. The reported sensitivity of ABR for the diagnosis of AN varies between 63 and 97%, however, for small AN, its sensitivity decreases significantly to 8–42% and ABR is not possible when the hearing loss exceeds 80 dB in the 2,000–4,000 Hz frequency (4, 38). A recent modeling study has found that the cost-saving with ABR prior to MRI does not seem to outweigh the number of missed patients with AN and other important pathologies that would have been detected when using standalone MRI (39). Therefore, for patients with SSNHL, we recommend that ABR and MRI should be combined to improve the accuracy of detection and prevent misdiagnosis and missed diagnoses, especially for small AN.

Previous studies have pointed out that the pathogenesis of SSNHL in patients with AN involves mechanical compression of the adjacent cochlear nerve, based upon the conjecture that the nerve fibers responsible for middle-frequency hearing are in a position more susceptible to tumor compression (14). Although hearing loss due to nerve compression is theoretically progressive, a sudden enlargement of the tumor (e.g., hemorrhage or cystic degeneration) could compress the cochlear nerve enough to cause sudden hearing loss (40). Nevertheless, it has been reported that tumor size is not to be correlated with the grade of hearing loss, and the correlation between tumor size and the incidence of SSNHL is also controversial (9, 16). In this study, we found no significant correlation between tumor size and the grade of hearing loss. In addition, the tumor size and configuration of audiograms were also unrelated. These results were consistent with other previous studies.

Many studies have observed that hearing recovery occurred in some patients with AN who presented with SSNHL after corticosteroid therapy and reported a recovery rate ranging from 16.7 to 44.4% (10, 30, 40). It is well-known that SSNHL patients with different types of audiogram configurations have obvious differences in their hearing recovery (1). Several studies have found that the recovery rate of SSNHL in patients with AN was also significantly related to audiogram patterns (26, 29). In 2021, Wasano et al. (16) revealed that the recovery of hearing in patients with U-shaped audiograms was significantly greater than in patients having the other audiogram forms, and the recovery rate decreased as the SSNHL episodes in patients increased. In 2017, Cho et al. (28) reported that non-tumorous lesions (intra-labyrinthine hemorrhage and labyrinthitis) showed a poorer treatment response than that of AN in patients with SSNHL. Hearing recovery may be due to the regression of tumor edema caused by corticosteroid treatment and/or the absorption of hemorrhage from the tumor itself or in the vicinity of the tumor (31). In the present study, 25% (2/8) patients with SSNHL diagnosed as AN showed hearing recovery after drug treatment. This rate was consistent with previous studies. These findings suggested that a therapeutic response to corticosteroid treatment for SSNHL does not exclude the presence of AN and all patients with SSNHL should undergo MRI to prevent misdiagnosis and delays in potential treatment.

## Conclusion

In conclusion, we have reported on a series of 10 patients with AN who presented SSNHL as a primary symptom and were treated as SSNHL initially. MRI is the most effective examination for the diagnosis of small AN. This study demonstrated that the hearing loss of these patients may improve with corticosteroid treatment. Therefore, we recommend that all patients presented with SSNHL, regardless of whether the hearing loss responds to drug treatment, should undergo MRI to rule out AN and avoid delayed treatment due to missed diagnosis. In addition, as an effective screening procedure, ABR is also important for the diagnosis of AN.

## Data availability statement

The original contributions presented in the study are included in the article/Supplementary material, further inquiries can be directed to the corresponding authors.

## Ethics statement

The studies involving human participants were reviewed and approved by the Committee of Medical Ethics of Chinese PLA General Hospital. The patients/participants provided their written informed consent to participate in this study.

## Author contributions

HW designed the work. DW provided data resources. JL, GC, and XZ acquired and analyzed data. MS and HW drafted, revised, and approved the manuscript. QW agree to be accountable for all aspects of the work. All authors have reviewed, discussed, and approved the manuscript.

## Funding

This work was supported by the grants of the National Key Research and Development Project (2020YFC2005200 and 2020YFC2005201); the National Natural Science Foundation of China (General Project 82171130); and the Analysis and Application of Medical Big Data in PLA General Hospital (2019MBD-005).

## Conflict of interest

The authors declare that the research was conducted in the absence of any commercial or financial relationships that could be construed as a potential conflict of interest.

## Publisher's note

All claims expressed in this article are solely those of the authors and do not necessarily represent those of their affiliated organizations, or those of the publisher, the editors and the reviewers. Any product that may be evaluated in this article, or claim that may be made by its manufacturer, is not guaranteed or endorsed by the publisher.

## Supplementary material

Supplementary Figure 1 Postoperative hearing results of the 4 patients who were treated with a surgical operation. Cases 3, 5, and 6 were taken with a translabyrinthine approach and case 7 with a retrosigmoid approach. A red triangle with an arrow was used for the right ear and a blue square with an arrow was used for the left ear to indicate that there was no response at maximum air conduction with masking.

Supplementary Table 1 Criteria for different configurations of audiograms.
