# Supplementary figures and images for: Sudden sensorineural hearing loss as the initial symptom in patients with acoustic neuroma
Source: Front Neurol. 2022 Aug 17;13:953265. doi: 10.3389/fneur.2022.953265 (PMC9430658; doi:10.3389/fneur.2022.953265)

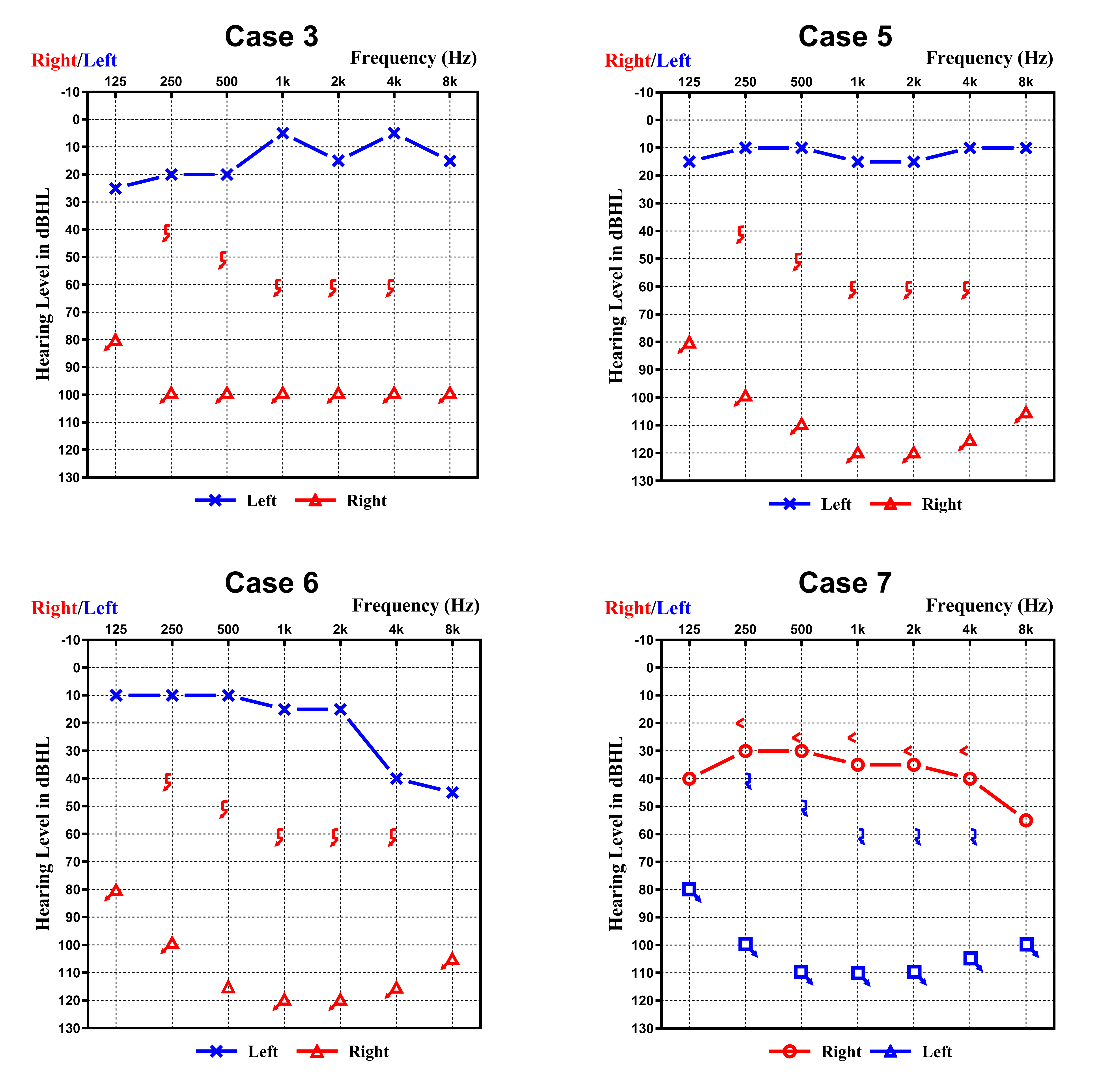

Supplement: Supplementary Figure 1 — Postoperative hearing results of the 4 patients who were treated with a surgical operation. Cases 3, 5, and 6 were taken with a translabyrinthine approach and case 7 with a retrosigmoid approach. A red triangle with an arrow was used for the right ear and a blue square with an arrow was used for the left ear to indicate that there was no response at maximum air conduction with masking. [file Image_1.TIF]
