# Supplementary material for: Sudden sensorineural hearing loss as the initial symptom in patients with acoustic neuroma
Source: Front Neurol. 2022 Aug 17;13:953265. doi: 10.3389/fneur.2022.953265 (PMC9430658; doi:10.3389/fneur.2022.953265)
Supplement: Supplementary Table 1 — Criteria for different configurations of audiograms. [file Table_1.DOCX]

| **Supplementary Table 1** Criteria for different configurations of audiograms | |
| --- | --- |
| Configurations of audiograms | Audiogram characteristics |
| Low-frequency ascending form | [(A + B + C) ÷ 3] – [(D + E + F + G) ÷ 4] > 15 dB |
| U-shaped form | (Poorest in C, D, and E) - (Poorest in A and B) > 15 dB  and (Poorest in C, D, and E) - (Poorest in F and G) > 15 dB |
| High-frequency descending form | [(F + G) ÷ 2] – [(D + E) ÷ 2] > 15 dB |
| Flat form | The difference between [(B + C) ÷ 2], [(D + E) ÷ 2], and [(F + G) ÷ 2] is < 15 dB |
| Profound form | Hearing thresholds are at ceiling levels at 3 or more frequencies in B, C, D, E, and F |
| Dip form | Does not meet any of the above criteria, and (Poorest in B, C, D, E, and F) - (all other frequencies) > 20 dB |
| Other form | The shape of the audiogram did not fit any of the six shapes above |

Abbreviations: Hearing threshold assessed at 0.125 kHz: A; Hearing threshold assessed at 0.25 kHz: B; Hearing threshold assessed at 0.5 kHz: C; Hearing threshold assessed at 1 kHz: D; Hearing threshold assessed at 2 kHz: E; Hearing threshold assessed at 4 kHz: F; Hearing threshold assessed at 8 kHz: G
